# Transformative Inclusive Education: A Case Study of an AI Game-Based Deep Learning Model for Literacy among Elementary School Students with Specific Learning Disorder (SLD)

**DOI:** 10.12688/f1000research.171885.1

**Published:** 2026-03-11

**Authors:** Minsih, Coiriyah Widyasari, Ratnasari Diah Utami, Imam Mujahid, Alfi Laila, Saiful Afandi, Hanum Salsabila

**Affiliations:** 1 Elementary Education, Universitas Muhammadiyah Surakarta, Surakarta, Central Java, Indonesia; 2 Universitas Muhammadiyah Surakarta, Surakarta, Central Java, Indonesia; 3 Universitas Muhammadiyah Surakarta, Surakarta, Central Java, Indonesia; 4 Universitas Islam Negeri Raden Mas Said Surakarta, Sukoharjo, Central Java, Indonesia; 5 Universitas Nusantara PGRI Kediri, Kediri, East Java, Indonesia; 6 Universitas Nusantara PGRI Kediri, Kediri, East Java, Indonesia; 7 Universitas Muhammadiyah Surakarta, Surakarta, Central Java, Indonesia

**Keywords:** Deep Learning, Literacy, Inclusion, AI Game, Specific Learning Disorder (SLD).

## Abstract

**Background:**

Inclusive students with Specific Learning Disorder (SLD) often experience persistent challenges in acquiring foundational literacy skills due to the lack of adaptive and personalized instructional approaches. Artificial Intelligence (AI) game-based deep learning models offer a promising alternative by enabling individualized learning pathways and continuous feedback. However, the integration of such models within inclusive primary school settings remains underexplored, particularly in Indonesia.

**Methods:**

This study employed a descriptive qualitative approach using a case study design to investigate the relevance and potential of AI game-based deep learning for literacy development among students with SLD. Data were collected through document analysis, review of scientific publications, and examination of related empirical studies. The collected data were analyzed using qualitative content analysis to identify key themes, conceptual patterns, and implications for inclusive literacy instruction.

**Results:**

The findings reveal three central insights. First, the adoption of AI game-based deep learning models is urgently needed to address the persistent literacy gaps among inclusive students with SLD. Second, such models demonstrate strong potential in supporting individualized literacy learning, particularly through features such as adaptive difficulty levels, automated feedback, and multimodal engagement. Third, the approach aligns well with the context of Indonesian primary schools, offering a feasible and pedagogically relevant tool for inclusive classrooms. The study also highlights the novelty of conceptualizing AI game-based deep learning not merely as a technological innovation but as a context-sensitive pedagogical model tailored to learners’ needs.

**Conclusions:**

AI game-based deep learning models hold significant promise for enhancing literacy outcomes among primary school students with SLD. Their adaptive and personalized nature provides meaningful support for inclusive education, helping to reduce learning barriers and promote equitable literacy development. Further research and pilot implementation are recommended to strengthen evidence-based adoption in schools.

## Introduction

Basic literacy skills are an important foundation for elementary school students to understand various subjects. ^1^ Good literacy supports students in thinking critically, communicating effectively, and accessing information independently. ^2^ However, not all students are able to master basic literacy easily, especially for inclusive students with specific learning disabilities (SLD). ^3^ This condition is exacerbated by the lack of learning approaches that are suitable for student characteristics. Therefore, a learning model that can respond to special needs and support students' overall literacy development is needed. The use of educational technology such as deep learning based on AI games can be a solution to create a more effective and adaptive learning experience. ^4^

Basic literacy skills in elementary school students serve as a crucial foundation for subsequent academic achievement. However, inclusive students with Specific Learning Disorder (SLD) often face significant challenges in mastering reading and writing skills due to difficulties in phonological processing, word recognition, or other disorders related to SLD. Recent studies indicate that traditional literacy interventions are often insufficient to meet the individual needs of students with SLD, thereby necessitating more personalized and responsive approaches. ^5^

Inclusive learning ideally provides space for every student, including those with SLD, to grow and develop without discrimination. Primary schools should be able to provide a learning system that supports students' diverse abilities. ^6^ Unfortunately, in practice, many SLD students have not received adequate support in the learning process, especially in literacy. Conventional methods are often unable to address students' individual needs, resulting in low learning outcomes. ^7^ Therefore, the integration of technology such as AI-based games with a deep learning approach can support learning that is more responsive to the needs of SLD students. ^8^ This model can provide real-time feedback and a fun and purposeful learning experience.

The use of technology in education can increase the effectiveness and efficiency of learning, including for students with special needs. Technology can provide a variety of media and approaches that strengthen student engagement in the learning process. ^9^ However, most digital learning platforms are still general in nature and pay little attention to the specific needs of SLD students. ^10^ The lack of personalized content is a major obstacle in improving students' literacy skills. ^11^ In this context, the use of deep learning models based on AI games is a promising solution to create adaptive and fun learning. Learning strategies utilizing this technology are tailored to the abilities and development of each student. ^12^

Digital technology, particularly deep learning and game AI, can be leveraged as an innovative solution to support personalized learning. Several studies have shown that this approach is able to provide real-time feedback, adapt materials to students' abilities, and increase learning motivation through gamification elements. ^1^ However, the utilization of this kind of technology in learning for SLD students is still very limited in the primary school environment, especially in Indonesia. ^7^

The advancement of artificial intelligence (AI) technologies has opened new opportunities for personalized education. AI has the capability to process large volumes of learning data and provide real-time feedback as well as adaptive learning materials tailored to each student’s profile. Within inclusive education contexts, AI-based tools have been reported to support early assessment, deliver adaptive learning recommendations, and enhance the accessibility of instructional materials for students with special needs. ^13, 14^

In parallel, game-based learning (GBL) has shown positive effects on motivation, engagement, and learning outcomes for early childhood and elementary students. Meta-analytic evidence and systematic reviews suggest that GBL can enhance both cognitive and affective aspects relevant to literacy development, such as attention, reading motivation, and enjoyable repeated practice. ^15, 16^ The integration of AI into GBL (AI-driven GBL) enables games to dynamically adjust the level of challenge, type of feedback, and learning pathways according to users’ responses Li. ^16^

AI-based educational games have great potential in increasing student engagement and motivation to learn. ^17^ When combined with deep learning technology, games can respond automatically to students' achievements and weaknesses in real time (Hendrianty et al., 2024). However, the adoption of AI-based games in learning is still not widespread in primary schools, especially in the context of inclusion. In fact, this approach can provide personalized and contextualized learning experiences for students with SLD. ^18^ To overcome this, there is a need for concrete efforts in developing and implementing learning models that systematically combine AI games and deep learning. ^19^ This can effectively encourage the strengthening of literacy in an inclusive primary school environment.

Innovation in learning models is needed to meet the challenges of 21st century education, especially for students with special needs. Learning models that use deep learning technology can help students understand material in a more in-depth and personalized way. ^20^ However, the application of such innovations is still limited due to the lack of understanding and training for educators in primary schools. ^21^ This causes delays in the adoption of new technology-based approaches. Therefore, it is important to improve educators' technological literacy and provide adequate infrastructure support. ^22^ Thus, the AI game-based deep learning model can be optimally applied to support the improvement of literacy skills of SLD students in primary schools.

Literacy learning in primary schools should be able to accommodate all students' diverse abilities, including students with specific learning disabilities. ^20^ However, the reality is that there are still many SLD students who struggle to access literacy materials due to the lack of adaptive learning approaches. ^23^ To overcome this, the application of AI game-based deep learning models can be a strategic solution that encourages personalization and effectiveness of literacy learning in an inclusive manner. ^18^

The research gap arises from the lack of studies that specifically explore the integration of deep learning models with AI games to support the literacy skills of inclusive students at the primary school level. Most of the previous studies only emphasize the benefits of technology in general or the development of educational applications without emphasizing personalization for special needs. Therefore, this article aims to examine the urgency, potential and relevance of using deep learning models based on AI games in improving the literacy skills of students with SLD in inclusive primary schools.

## Methods

This research uses a descriptive qualitative approach with a literature study method to analyze the urgency and potential of using an AI game-based deep learning model in improving the literacy skills of inclusive students with Specific Learning Disorder (SLD) in elementary schools. This approach was chosen because it is suitable for studying phenomena based on secondary data in the form of published literature and scientific documents. ^23^

**Figure 1.**
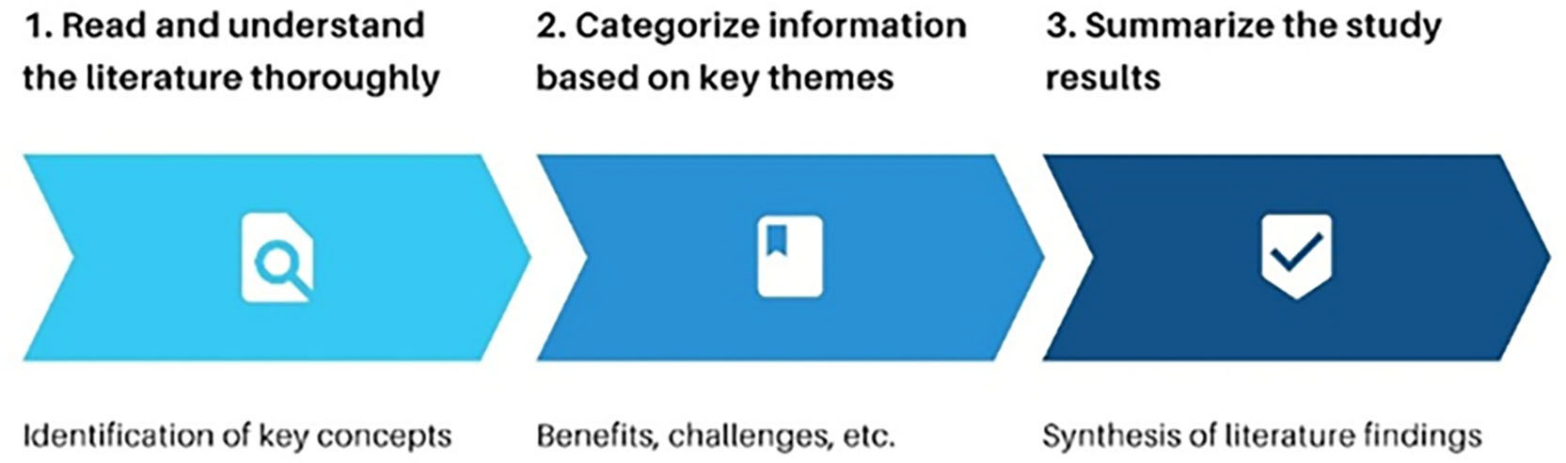
Qualitative content analysis stage.

Data sources were obtained through a systematic search of relevant national and international journals, reference books, and research reports from 2020 to 2025. The keywords used in the search included deep learning, AI games, inclusive student literacy, Specific Learning Disorder (SLD), and inclusive primary education. Sources were selected based on the credibility of the publisher and the relevance of the topic to the research focus. Data analysis was conducted using the qualitative content analysis method, which aims to identify patterns, concepts, and important findings from various literatures (Wahyuni et al., 2023). This process includes three main stages, namely:

1. **Reading and Understanding**
  Thoroughly review each literature source to understand the key concepts, terminology and arguments related to AI technology and inclusive learning.
2. **Categorizing Information**
  Organize information into key themes based on issue focus, such as technology benefits, implementation challenges, and literacy strategies.
3. **Summarizing the Study Results**
  Summarize the findings in the form of a thematic synthesis that is relevant to the research objectives. The following are the stages of qualitative content analysis (see Figure 1).

Description:

Step 1: Focus on literature exploration and context understanding

Step 2: Information is categorized

Step 3: Review is summarized

This study employed a **descriptive qualitative approach** with a literature study method to analyze the urgency and potential of an AI game-based deep learning model in improving the literacy proficiency of inclusive students with Specific Learning Disorder (SLD) in elementary schools. This approach was selected because it is well suited for examining phenomena based on secondary data drawn from published academic sources. ^23^

Data sources were systematically collected from February to June 2025 through national and international academic databases. The keywords used in the search included *deep learning*, *AI games*, *inclusive student literacy*, *Specific Learning Disorder (SLD)*, and *inclusive primary education.* Documents included peer-reviewed journal articles, reference books, and research reports published between 2020 and 2025. To verify the integrity of data, only peer-reviewed and officially published sources were included, while duplicate or unreliable documents were excluded. All documents were stored digitally in a secure repository, accompanied by metadata (author, year, source type, and thematic category), and managed with version control to ensure consistency and reliability.

Since this research relied solely on secondary data, no direct involvement of human participants occurred. However, ethical research standards were maintained by:

1. Respecting intellectual property rights through proper citation and referencing.
2. Ensuring accuracy and integrity in the interpretation of sources.
3. Avoiding plagiarism and misrepresentation of findings.
4. Transparently reporting the procedures of data collection, management, and analysis.

The data in this study were analyzed using **qualitative content analysis**, which is suitable for systematically interpreting patterns, concepts, and themes from textual data. ^14, 24^ The process of analysis followed three phases: **open coding, axial coding, and selective coding.** This stepwise approach ensured that the interpretation was grounded in the data and aligned with the research objectives. In the **open coding** phase, the researcher carefully read and re-read the selected documents to identify key concepts and assign initial codes. For instance, terms such as *phonological difficulty*, *word recognition issues*, *AI adaptation*, and *student motivation* emerged as early codes. These codes captured meaningful segments of text that were relevant to literacy learning challenges, AI-based personalization, and game-based engagement. During the **axial coding** phase, similar codes were clustered into broader categories. For example, “phonological difficulty” and “word recognition issues” were grouped into the category *literacy barriers*, while “AI adaptation” and “real-time feedback” were clustered into *AI-based personalization.* In the final **selective coding** phase, categories were integrated into central themes that directly addressed the research questions. For instance, *literacy barriers* and *AI-based personalization* were synthesized into the theme *the role of AI game-based deep learning in overcoming literacy challenges for SLD students.* This process allowed the researcher to move from fragmented codes to holistic themes.

To demonstrate the analytic process, a coding framework was developed (see Table 1). This framework shows the relationship between initial codes, axial categories, selective themes, and source excerpts, thereby providing transparency and traceability of the analysis (see Table 1).

**Table 1.** Framework of data coding and synthesis.

Code (Initial)	Category (Axial)	Theme (Selective)	Example source extract
Phonological difficulty	Literacy barriers	Barriers to literacy learning for SLD students	“SLD students struggle in recognizing phonemes …”
Word recognition issues	Literacy barriers	Barriers to literacy learning for SLD students	“Difficulty in identifying sight words …”
AI adaptation	AI-based personalization	Role of AI in customizing learning paths	“AI can provide adaptive feedback …”
Real-time feedback	AI-based personalization	Role of AI in customizing learning paths	“The model adjusts the task difficulty …”
Student motivation	Game-based engagement	Effect of game-based learning on student engagement	“Game design fosters motivation and attention …”
Repeated practice	Game-based engagement	Effect of game-based learning on literacy development	“Repetition embedded in gameplay improves reading fluency …”
Inclusive classroom fit	Contextual implementation	Relevance of AI game-based models in inclusive primary education	“Model tested in inclusive schools in Indonesia …”

To strengthen the rigor of the analysis, **NVivo 14 software** was employed. NVivo supported the management of large sets of documents, enabled systematic coding, facilitated the merging and refinement of categories, and provided visualization tools (such as cluster analysis and models) to examine relationships among themes. Moreover, NVivo maintained an audit trail of all coding activities, which increased transparency and allowed replication of the analytic process. To ensure the integrity of the data, only **peer-reviewed and officially published documents** were included in the dataset, while duplicates and unreliable sources were excluded. All documents were stored digitally in a secure repository and labeled with metadata (author, year, type of source, thematic category). Version control was applied to track updates and revisions, thereby ensuring consistency and reliability throughout the research process.

Furthermore, **triangulation** was conducted to enhance the trustworthiness of findings. This study applied:

1. **Source triangulation**, by comparing data from different types of documents (journal articles, books, and research reports) and across various databases, to minimize bias from a single source.
2. **Methodological triangulation**, by combining systematic literature review procedures with qualitative content analysis to cross-validate emerging categories and themes.
3. **Theoretical triangulation**, by interpreting findings using multiple relevant theoretical frameworks, such as learning theory, inclusive education principles, and AI-based instructional design models.

Through triangulation, data consistency was verified, discrepancies were critically examined, and the credibility of the analytic results was reinforced.

This research aims to develop a strong conceptual basis for the application of AI-based learning technology in inclusive education, as well as an initial reference for further research that wants to develop or test AI game-based deep learning models directly in the field.

## Result

### Stage 1: Reading and understanding

The initial stage was conducted by thoroughly reading and understanding scientific sources relevant to the topic of literacy learning for inclusive students with SLD using deep learning technology based on AI games. This process involved an in-depth exploration of international journal articles, academic books and research reports from the last five years curated based on the credibility of the publisher and relevance to the focus of the study. All documents were actively read to identify terminology, conceptual frameworks and key arguments related to the use of AI technology in inclusive primary education. In addition, the search focused on the context of technology intervention in literacy-based learning and its impact on the cognitive development of students with special needs. It was found that most of the studies emphasized the importance of personalized learning that is responsive to students' characteristics. This thorough reading process formed the basis for further data clustering and analysis in the next stage. However, there are not many studies that explicitly discuss the integration of deep learning and game AI in the context of literacy education for SLD students, especially at the primary school level in Indonesia. This finding becomes an important basis for the information clustering process in the next stage.

### Stage 2: Categorizing information

After the reading and understanding stage was completed, the information obtained was grouped into main themes that reflected the direction of the findings and the focus of the problem being analyzed. This grouping was done to formulate a systematic structure of thought on the issues and potential of AI game-based deep learning models in the context of literacy learning in inclusive primary schools. Each theme is designed to represent the relationship between technological approaches and meeting the needs of students with specific learning barriers in the primary school environment. A visualization of the results of this grouping is shown in Table 1, which includes three major themes: the benefits of deep learning technology in inclusive learning, the potential of AI games as a literacy medium, and the challenges and strategic recommendations in implementing the model (see Table 2).

**Table 2.** Information grouping results.

Theme	Key findings	Direct excerpts	Supporting sources
Benefits of Deep Learning in Inclusive Learning	1. Automatically detect patterns of learning difficulties. 2. Provide adaptive learning based on student progress. Supports real-time material differentiation.	“Deep learning technology provides real-time adaptation to student interaction, allowing materials to be adjusted to their individual learning pace.”	1. (Hendrianty et al., 2024) 2. (Adriana, 2021) 3. (Nugroho et al., 2025) (Hidayah & Suyitno, 2021)
The Potential of AI Games as Literacy Learning Media	1. Increase active participation and motivation of SLD students. 2. Adjust the level of challenge based on student performance. 3. Facilitate fun learning through reward system.	“AI-based educational games motivate students through instant feedback and gamified rewards that sustain engagement.”	1. (Nurviyani et al., 2024) 2. (Maufidhoh & Maghfirah, 2023) 3. (Auer & Centea, 2021) 4. (Wahyuni et al., 2023)
Implementation Challenges and Strategic Recommendations	1. Limited digital infrastructure in primary schools. 2. Low technology literacy of teachers. 3. The curriculum does not explicitly support AI integration.	“Teachers’ limited digital literacy remains a critical barrier in adopting AI-based learning models.”	1. (Iqbal, 2025) 2. (Nikadinata et al., 2025) 3. (Hartatik et al., 2023) (Linawati et al., 2024)

### Theoretical and practical contributions

1. **Theoretical Contribution**
  This study contributes theoretically by enriching the conceptual framework of technology-based inclusive learning. The AI game-based deep learning model emphasizes that real-time personalization can serve as a new foundation for differentiated learning theories. Furthermore, this study reinforces the perspective that technology integration is not merely a supplementary tool but a conceptual framework capable of addressing the specific needs of students with Specific Learning Disorder (SLD).
2. **Practical Contribution**
  Practically, the findings of this study are useful in three major aspects:
  a. For teachers: providing a basis for designing adaptive literacy learning tailored to the characteristics of SLD students.
  b. For schools and policymakers: offering recommendations to strengthen digital infrastructure and teacher training in implementing AI game-based learning.
  c. For educational technology developers: serving as a reference in creating literacy applications that are engaging, adaptive, and responsive to the needs of inclusive students.

The table above illustrates how information from various sources has been thematically classified to clarify the position of the AI game-based deep learning model in supporting SLD students' literacy. Each theme reflects a different aspect of this model, ranging from the power of technology in customizing learning materials, the potential of games as effective pedagogical instruments, to various challenges that need to be considered in the context of real implementation. This classification process not only helped to build a solid conceptual framework, but also served as the basis for synthesizing and reflecting on the overall findings. By bringing together conceptual and contextual aspects, this analysis provides a comprehensive insight into the relevance and feasibility of the learning model. In addition, this clustering process shows that technology cannot stand alone without policy support and adequate educator competencies. The results also emphasize that literacy education in inclusive primary schools requires the support of learning technologies that are not only sophisticated but also contextual to the characteristics of early childhood students and the primary level curriculum.

The table outlines how AI-based approaches have great potential in supporting inclusive literacy. However, there is no research in Indonesia that has developed and tested a deep learning-based AI game model specifically designed for SLD students at the primary school level. This indicates an important research gap that needs to be bridged through local innovation based on the needs of the Indonesian inclusive education context.

### Stage 3: Summarizing the study results

After the process of reading and categorizing the information, a process of summarizing the results of the study was carried out, which aimed to integrate all the findings into a reflective framework for the research objectives. This summarization was organized thematically based on the results of the previous classification and focused on three main aspects: the urgency of applying the model, the transformational potential of this approach and its relevance in the context of inclusive primary education. A full description of the synthesis of these three aspects follows.

The summarization process was conducted by integrating the findings from the previous two stages into three main focuses:

1. The urgency of implementing AI game-based deep learning model in the context of SLD students,
2. The transformational potential of this approach to literacy learning,
3. Contextual relevance in Indonesia's inclusive primary education policy.

The results of this study build a strong conceptual foundation for further applied research and encourage cross-sector collaboration to address the need for inclusive smart technology-based literacy.

1. **Urgency of Implementing AI Game-Based Deep Learning Model**

The deep learning-based learning model integrated in AI games shows high urgency to be implemented in an inclusive elementary school environment. SLD students in elementary schools who have unique learning characteristics need a learning system that is not uniform, but can adjust to the speed and way of learning of each individual. Deep learning technology provides the flexibility needed as it is able to analyze student interaction data in real-time and adjust learning content automatically. This makes the learning process more effective and efficient, and increases students' chances of understanding literacy materials more deeply. In the context of conventional learning, challenges such as time constraints and rigid learning methods often hinder the progress of SLD students. Therefore, the need for this kind of learning model is not only urgent, but also strategic in supporting the inclusiveness of education in real terms.

2. **Transformational Potential of AI Game Approach in Literacy Learning**

The application of AI games in the context of literacy learning offers significant transformational potential in improving the motivation and learning engagement of students with SLD. Through the gamification approach, students experience learning in a more fun, less intimidating and curiosity-provoking atmosphere. The adaptive gaming system also provides space for students to try, fail and relearn independently without social or academic pressure. In addition, the elements of healthy competition and instant feedback present in AI-based games help students gain immediate reinforcement for their achievements. This potential is even stronger when combined with the AI's ability to analyze performance and adjust challenge levels accordingly. This approach is particularly relevant in the context of literacy learning at the primary school level, which not only impacts cognitive aspects, but also encourages emotional and social aspects in the learning process. Therefore, AI games are not just learning aids, but also a learning system that can revolutionize the way students access literacy.

3. **Relevance of the Model in the Context of Inclusive Primary Education**

The AI game-based deep learning model has high relevance in the context of literacy learning in inclusive primary schools in Indonesia. The integration of this technology is also in line with basic education policies that emphasize the importance of differentiation and personalization of learning. However, implementation in the field still faces various obstacles such as the lack of technological infrastructure, limited training for educators, and the lack of curriculum that accommodates this approach explicitly. Therefore, this model needs to be introduced gradually through a pilot program that involves collaboration between the government, schools and the education technology community. In addition, there needs to be more massive advocacy on the importance of innovation in inclusive education so that this approach receives broad support from policy makers and the community. The relevance of this model lies not only in its technological aspects, but also in its capacity to bridge the gap in access and quality of learning for students with special needs.

The AI game-based deep learning model has promising theoretical and practical strengths for implementation in inclusive primary education systems. This analysis shows that the approach is not only innovative but also responsive to the real challenges SLD students face in literacy acquisition. Although various implementation challenges still need to be overcome, the findings affirm the new direction of inclusive and smart technology-based education. The role of teachers as facilitators is crucial in translating technological sophistication into meaningful learning practices. Policy support, intensive training and cross-sector collaboration will determine the success of this approach. With proper development, this model has a great opportunity to become the foundation of the transformation of inclusive education based on equity and accessibility.

## Discussion

### 1. Urgency of implementing AI game-based deep learning model

The findings of this study confirm the necessity of implementing an AI game-based deep learning model in inclusive primary schools. Students with Specific Learning Disorder (SLD) require adaptive approaches to engage effectively in literacy learning. Consistent with, ^18, 23, 25^ the results demonstrate that deep learning technology can personalize content delivery in real time according to students’ unique patterns of learning, and the use of strategies can provide benefits for student with SLD. Moreover, the evidence supports earlier studies by, ^22, 26, 27^ which argued that conventional uniform methods are insufficient for inclusive classrooms. Task adjustments and additional time provided enable slow learners to perform better on assignments. The arrangement of the learning environment, including seating placement and positive feedback, also plays a crucial role in creating a conducive and supportive learning atmosphere. In this respect, our study both confirms prior conclusions and advances them by emphasizing the synergy between deep learning algorithms and game-based pedagogy as a unified model. Unlike previous work, which often analyzed either AI tools or gamification separately, this study highlights their combined potential to create more responsive and inclusive learning environments.

### 2. Transformational potential of AI game approach in literacy learning

The results further demonstrate that AI game-based deep learning holds strong transformational potential for literacy acquisition. Previous studies ^7, 8, 28^ highlighted the benefits of gamification and instant feedback in increasing student engagement. Our findings not only confirm these benefits but also extend them by showing that when combined with deep learning technology, gamification can dynamically adjust challenge levels and learning pathways based on real-time analytics. This advancement contrasts with earlier approaches, which primarily relied on static gamified modules without adaptive intelligence. In this way, the present study offers an improvement over prior research by proposing an integrative design that ensures both engagement and personalization simultaneously.

### 3. Relevance of the model in the context of inclusive primary education

The AI game-based deep learning model has demonstrated high relevance for inclusive education in Indonesia, aligning with differentiation and personalization principles emphasized in international inclusive learning frameworks. ^12, 17^ This study improves upon earlier work ^19, 29, 30^ by contextualizing these principles within the specific challenges of Indonesian primary schools, where infrastructure and teacher capacity remain critical barriers.

While prior research has acknowledged such constraints in general terms, our study contributes a clearer **practical roadmap** for addressing them:

a. **Pilot Project Design**: Conducting classroom-based trials in selected inclusive schools to test the feasibility, scalability, and adaptability of the model.
b. **Integration into National Literacy Curriculum**: Aligning the AI game-based deep learning framework with Indonesia’s literacy and inclusive education policies to ensure long-term sustainability.
c. **Teacher Professional Development**: Designing systematic training programs to enhance teachers’ technological literacy, enabling them to integrate AI tools effectively into everyday instruction.

This roadmap provides a practical guide for policymakers, practitioners, and stakeholders to move beyond conceptual adoption toward sustainable implementation.

### 4. Theoretical contribution and novelty

The study explicitly introduces a **conceptual framework of AI game-based deep learning for inclusive literacy education**. This framework contributes theoretically by demonstrating how real-time personalization through deep learning algorithms can be integrated with gamification principles to address diverse literacy challenges among SLD students. The novelty lies in the **integration of two domains deep learning and AI-based games into a single adaptive model specifically tailored for inclusive primary schools**. To our knowledge, no prior research in the Indonesian context has systematically conceptualized and synthesized these elements into a unified pedagogical framework. This represents both an advancement in theoretical discourse on differentiated education and a unique contribution to the body of inclusive learning scholarship.

### 5. Global relevance of findings

Although the present study is rooted in Indonesia, the implications extend to broader educational contexts, particularly in developing countries. Many nations across Asia, Africa, and Latin America face similar challenges limited infrastructure, uneven teacher training, and insufficient curriculum adaptation for inclusive education. The proposed AI game-based deep learning framework can serve as a scalable model for these contexts by offering adaptive, resource-efficient, and engaging literacy solutions. Its emphasis on personalization and gamified motivation ensures that the model is not bound to a single cultural or institutional context, but rather can inform global efforts toward achieving equitable literacy in inclusive education systems.

## Conclusion

This study concludes that the AI game-based deep learning model holds urgent and strategic relevance for inclusive primary education, particularly for students with Specific Learning Disorder (SLD). The findings not only support previous studies emphasizing the importance of personalization in inclusive learning, ^18, 31^ but also extend them by demonstrating the integrative power of deep learning algorithms and gamification elements within a single pedagogical model. Unlike earlier approaches that examined either artificial intelligence or game-based methods in isolation, this research provides a unified conceptual and practical framework for adaptive literacy instruction.

The integration of gamification makes literacy learning more engaging, interactive, and pressure-free, while deep learning mechanisms enable real-time personalization based on students’ interaction data. Nevertheless, several limitations remain, such as the risk of student overreliance on games, potential bias in AI systems, and challenges related to infrastructure and teacher competence. Addressing these limitations requires a practical roadmap, including: a) the development of teacher training modules that enhance educators’ technological literacy for AI-supported instruction, b) multi-stakeholder collaboration among schools, government agencies, and educational technology startups, and c) gradual pilot implementation in selected inclusive schools prior to large-scale adoption.

The contribution of this research is twofold. Theoretically, it proposes a novel conceptual framework that integrates deep learning and gamification to meet the diverse literacy needs of SLD students in inclusive settings. Practically, it offers actionable recommendations for Indonesian policymakers to align this model with the nation’s inclusive education agenda, thereby supporting equitable access to literacy learning as outlined in national education policies.

Beyond Indonesia, the lessons learned from this study are highly relevant for other developing countries facing similar challenges in digital infrastructure, curriculum adaptation, and teacher readiness. By adopting context-sensitive AI game-based deep learning models, nations across Asia, Africa, and Latin America can advance inclusive literacy education while moving toward global commitments to Sustainable Development Goals (SDG 4: Quality Education).

In summary, while acknowledging the research gap in the absence of AI game models tailored for SLD students in Indonesia, this study contributes uniquely by offering both a conceptual innovation and a practical implementation pathway. This dual contribution not only enriches the theoretical discourse on differentiated and inclusive education but also provides a foundation for cross-national application of AI-driven literacy solutions.

## Ethical considerations

This study employed a systematic literature review methodology and did not involve human participants, animals, or primary data collection. All data were obtained from publicly available, peer-reviewed publications. Therefore, ethical approval and informed consent were not required. The review process was conducted in accordance with principles of academic integrity, including proper citation, transparency in data selection, and avoidance of plagiarism.

## Data Availability

All data supporting the findings presented in this article are openly accessible through public repositories under a Creative Commons Attribution (CC-BY) license. The dataset includes numerical values used in the calculation of means, standard deviations, and various other statistical metrics essential to the analysis. Raw data underlying tables, figures, and graphical representations are also provided, along with extracted data points used for subsequent analytical procedures. Making these data publicly available is intended to enhance scientific transparency, facilitate verification and replication by other researchers, and promote broader data utilization for further studies in related fields. This open-data approach strengthens the rigor, accountability, and overall contribution of the research to the advancement of scientific knowledge. Figshare: Manuscript Data AI Based Deep Learning. SLR Coding Matrix Grouping Summary. DOI: https://doi.org/10.6084/m9.figshare.30987778 ^32^ Data are available under terms of the Creative Commons Attribution 4.0 International license (CC-BY 4.0).
