# Supplementary material for: Transformative Inclusive Education: A Case Study of an AI Game-Based Deep Learning Model for Literacy among Elementary School Students with Specific Learning Disorder (SLD)
Source: F1000Res. 2026 Mar 11;15:391. [Version 1] doi: 10.12688/f1000research.171885.1 (PMC13182786; doi:10.12688/f1000research.171885.1)
Supplement: Peer review history

# Reviewer report: Reviewer response for version 1

**DOI:** 10.5256/f1000research.189547.r468729

**Published:** 2026-04-03

**Authors:** Resti Yektyastuti

**Affiliations:** 1 Universitas Djuanda, Bogor, Indonesia

**Summary of the Article**

 This manuscript explores the potential of an AI game-based deep learning model to support literacy development among elementary school students with Specific Learning Disorder (SLD) in inclusive education settings. The study employs a qualitative literature-based approach to synthesize existing research and proposes a conceptual framework integrating artificial intelligence, gamification, and adaptive learning.

 The topic is highly relevant, particularly in the context of inclusive primary education, where early literacy support for students with SLD is essential. The manuscript is generally well-structured and provides useful conceptual insights and practical implications for teachers and policymakers.

**1. Clarity of presentation and use of literature (Partly)**

 The manuscript is clearly written and logically organized. However, the use of literature could be strengthened. A considerable proportion of references are drawn from local or regional sources. Increasing the use of recent studies from reputable international journals would enhance the academic rigor and global relevance of the manuscript. Strengthening connections with established frameworks in inclusive and special education would further improve the discussion.

**2. Study design and technical soundness (Yes)**

**3. Methods and replicability (Partly)**

 The manuscript outlines the general process of literature review and qualitative content analysis. However, additional detail would improve transparency and replicability. Clarification regarding the number of sources reviewed search procedures, inclusion criteria, and coding process would strengthen methodological clarity.

**4. Statistical analysis (Partly)**

 The study is primarily qualitative and does not explicitly present statistical analysis within the main manuscript. However, the data availability section refers to numerical values and statistical metrics, suggesting that some quantitative elements may be involved. The role and interpretation of these elements are not clearly explained or integrated into the analysis. Clarifying whether and how these data contribute to the findings would improve consistency and transparency.

**5. Data availability and reproducibility (Partly)**

 The authors provide access to supporting data through a public repository, which is commendable. However, the description of the dataset could be clarified further. Providing a clearer explanation of what the dataset contains (e.g., literature extraction or coding results) would improve alignment with the qualitative approach and enhance reproducibility.

**6. Validity of conclusions (Yes)**

**Points that Should Be Addressed**

1. Enhance the quality and international scope of references, particularly by incorporating more recent studies from reputable journals.
2. Provide clearer methodological details regarding literature selection and analysis.
3. Clarify the nature of the dataset to ensure consistency with the qualitative approach.
4. Clarify the mention of statistical elements to improve coherence across sections.

Overall, this manuscript offers a valuable perspective on inclusive education and emerging technologies. With some refinements, it can provide an even stronger contribution to both research and practice.

Is the work clearly and accurately presented and does it cite the current literature?

Partly

If applicable, is the statistical analysis and its interpretation appropriate?

Partly

Are all the source data underlying the results available to ensure full reproducibility?

Partly

Is the study design appropriate and is the work technically sound?

Yes

Are the conclusions drawn adequately supported by the results?

Yes

Are sufficient details of methods and analysis provided to allow replication by others?

Partly

Reviewer Expertise:

Elementary school, special education in elementary school, learning media for special education, science education in elementary school

I confirm that I have read this submission and believe that I have an appropriate level of expertise to confirm that it is of an acceptable scientific standard, however I have significant reservations, as outlined above.

# Reviewer report: Reviewer response for version 1

**DOI:** 10.5256/f1000research.189547.r468735

**Published:** 2026-04-03

**Authors:** Lorena Jaramillo-Mediavilla

**Affiliations:** 1 Technical University of the North (Ringgold ID: 27909), Ibarra, Imbabura Province, Ecuador

The manuscript addresses a relevant, timely, and important topic in inclusive education by proposing the integration of artificial intelligence, gamification, and deep learning to strengthen literacy among primary school students with Specific Learning Disorder (SLD). The article offers a valuable conceptual contribution and raises a meaningful issue in the Indonesian context, where the integration of this type of model remains limited. Overall, the work has thematic merit and cites recent literature; however, it requires substantial revisions to achieve greater scientific rigor.

 First, although the manuscript is understandable and follows a recognizable academic structure, its clarity is weakened by inconsistencies in the definition of the methodological design. In the abstract, it is presented as a “case study,” whereas in the Methods section it is described as a descriptive qualitative study based on a literature review, and in the Ethical Considerations section it is referred to as a systematic literature review. These designations are not equivalent and create ambiguity regarding the true nature of the study. This issue should be corrected so that there is full coherence between the title, abstract, methods, and conclusions.

 Second, the study design may be appropriate for building a conceptual framework, but in its current form it is not fully technically sound. The article does not present empirical evidence obtained through intervention, piloting, or direct application with students with SLD; rather, it relies on document analysis and thematic synthesis of the scientific literature. Therefore, the findings should be interpreted as a conceptually grounded proposal rather than as an empirical validation of the model. Consequently, several statements in the discussion and conclusions should be moderated, especially those related to the model’s effectiveness, feasibility, or transformative capacity.

 Third, although the Methods section mentions systematic searching, qualitative content analysis, open, axial, and selective coding, triangulation, and the use of NVivo 14, it still lacks sufficient detail to allow rigorous replication of the study. It would be necessary to specify more precisely the databases consulted, the full search strategy, the initial and final number of documents included, the inclusion and exclusion criteria, and the procedure through which the selected documents were transformed into the final categories and themes. Without this information, the reproducibility of the study remains only partial.

 Fourth, the Data Availability section presents an important inconsistency with the type of study reported. Although the article is defined as a qualitative literature-based study, the data section mentions numerical values, means, standard deviations, and other statistical metrics. This description does not appear to correspond to the actual design of the manuscript and should be revised. The authors should clarify exactly what the repository contains, for example, a coding matrix, the analyzed document corpus, thematic categories, analytic memos, or other qualitative materials. As currently written, the data availability statement does not ensure full reproducibility in a way that is fully consistent with the declared methodological approach.

 Fifth, the conclusions are generally aligned with the documentary review that was conducted, but in several passages they go beyond the level of evidence actually available. The article makes relatively strong claims regarding the strategic relevance, transformative potential, and broad applicability of the proposed model, including for other international contexts. Since there is no direct empirical validation, it would be more rigorous to rephrase these statements more cautiously, indicating that the study suggests, conceptually supports, or proposes a framework for future validation in real-world contexts.

 In summary, I consider that the manuscript has conceptual value, thematic relevance, and publication potential, but it requires major revisions in order to become scientifically stronger. In particular, the authors should:

 (1) clearly and consistently define the type of study conducted;

 (2) strengthen the methodological description to improve replicability;

 (3) revise the Data Availability section so that it is coherent with a qualitative study based on documentary review;

 (4) moderate the scope of the claims made in the discussion and conclusions; and

 (5) review the editorial and terminological consistency of the manuscript.

 My overall assessment is that the article has an interesting conceptual basis, but it still requires important adjustments in methodological coherence, argumentative precision, and analytical traceability before it can be considered fully robust from a scientific standpoint.

Is the work clearly and accurately presented and does it cite the current literature?

Partly

If applicable, is the statistical analysis and its interpretation appropriate?

Not applicable

Are all the source data underlying the results available to ensure full reproducibility?

Partly

Is the study design appropriate and is the work technically sound?

Partly

Are the conclusions drawn adequately supported by the results?

Partly

Are sufficient details of methods and analysis provided to allow replication by others?

Partly

Reviewer Expertise:

Inclusive education; literacy education; educational technology; AI in education; game-based learning; qualitative research methods; teacher development in primary education.

I confirm that I have read this submission and believe that I have an appropriate level of expertise to confirm that it is of an acceptable scientific standard, however I have significant reservations, as outlined above.

# Reviewer report: Reviewer response for version 1

**DOI:** 10.5256/f1000research.189547.r468728

**Published:** 2026-04-03

**Authors:** Ivana Roncevic

**Affiliations:** 1 University of Slavonski Brod, Slavonski Brod, Croatia; 2 University of Zagreb, Zagreb, Croatia

**Summary**

 The article presents a case study investigating how AI game-based deep learning can be employed for individualized learning approaches supporting primary students with Specific Learning Disorder (SLD) in Indonesia. The authors apply a descriptive qualitative approach to relevant studies on the topic published between 2020 and 2025. The findings reveal that students with SLD are in urgent need of support in literacy development and that the AI game-based deep learning approach can support individualized literacy learning, as it can adapt for difficulty levels, provide automated feedback and engage students in a multimodal way. Authors also emphasize the attractiveness of the approach due to its context-sensitive pedagogical model adapted to learners’ needs.

 The study presents an ambitious and timely topic, but several concerns regarding study design, methodological rigor, replicability, and the strength of conclusions should be addressed.

 The paper follows a logical structure with clearly defined stages, a coding framework and themes. The references are timely and relevant. The choice of a descriptive qualitative approach with a systematic literature review methodology is broadly appropriate for a study at this conceptual and exploratory stage. Mapping the landscape of AI-based tools for SLD literacy, particularly in a context as underexplored as Indonesian inclusive primary education, warrants a literature-based foundation before empirical trials are undertaken. The use of NVivo 14 and triangulation strategies (source, methodological, and theoretical) also reflects an awareness of rigor. The authors provide a general description of their three-phase content analysis process and reference NVivo 14 as their analytical tool. Table 1 offers a useful coding framework with example source excerpts. The conclusions are logically coherent and internally consistent with the themes derived from the literature synthesis. The practical roadmap with pilot project design, curriculum integration, and teacher professional development is sensible. 

**The authors should revise the paper in the following aspects: **

1. The title and abstract do not reflect the real nature of the study, which is a literature review and not a case study.
2. PRISMA framework with exclusion and inclusion criteria should be included. 
3. The term “deep learning” is not used fully in accordance with the literature. Throughout the paper, it is used in two distinct senses — the Indonesian Ministry of Education's pedagogical framework ("Pembelajaran Mendalam") and the machine learning technique involving neural networks. These are categorically different concepts, and the authors need to clearly and consistently disambiguate them.
4. Regarding the cited literature, it is advisable to expand the geographical and linguistic scope of the reviewed sources. Authors are advised to include internationally cited sources in English, which can be verified by international audience. A disproportionate share of cited works are Indonesian-language publications from journals with limited international visibility. While local context is important, a study making claims about global relevance and applicability to Asia, Africa, and Latin America must engage more substantively with the international literature on AI in special education, dyslexia and SLD intervention research, and adaptive learning systems.
5. The authors make several key claims about deep learning and game-based learning, but to support those claims, they rely on general sources rather than specific ones. It is advised to find more specific literature sources to support those claims. The paper would benefit from engaging more directly with prominent international literature on AI in special education and dyslexia research specifically. The foundational work in those areas is underrepresented.
6. There is repetition in the text, for example in methodology. Empirical claims should be separated from conceptual arguments. 
7. Replicability: The study needs to ensure that it can be replicated by other researchers. To ensure that, it needs to provide the complete list of analyzed sources. The reference list contains 32 items, but it is unclear which subset of these constituted the actual analytical corpus versus background reading. A dedicated appendix listing all documents included in the content analysis, with metadata (author, year, source type, database, thematic category), is advised.
8. The coding process: The process named in the study, consisting of three phases, is briefly described. What should be documented: the number of initial codes generated, the process by which codes were merged or refined, and the decision rules applied. Also, inter-rater reliability should be reported. It should be made clear of whether multiple coders independently coded the same documents, whether disagreements occurred, or how they were resolved. 
9. Statistics: The data availability statement mentions "means, standard deviations, and various other statistical metrics”. This is not in alignment with the actual study, which is a qualitative literature review. The authors should clarify or correct this statement. 
10. The authors provide a Figshare repository to the data. However, full reproducibility requires the complete analyzed corpus to be listed and accessible.
11. Conclusion: The paper seems to make claims which are not supported by strong evidence. When the authors conclude that AI game-based deep learning models "hold urgent and strategic relevance" and offer "significant promise" for SLD literacy outcomes, there should be more than conceptual information to support those claims. As this is not an empirical study, the conclusions should reflect that reality. What they can claim is that the theoretical case for such models is promising, and that empirical investigation is warranted.
12. The limitations section is brief and somewhat underdeveloped. Critical limitations that should be explicitly acknowledged include: the absence of empirical data; the risk of confirmation bias in source selection; the heavy reliance on Indonesian-language and potentially lower-impact sources; and the absence of any implemented model to evaluate. 
13. The authors assert that no prior research in Indonesia has systematically integrated deep learning and AI-based games into a unified pedagogical framework for SLD students. It is advisable to also mention whether such research has been done internationally.

Is the work clearly and accurately presented and does it cite the current literature?

Partly

If applicable, is the statistical analysis and its interpretation appropriate?

Not applicable

Are all the source data underlying the results available to ensure full reproducibility?

Partly

Is the study design appropriate and is the work technically sound?

Partly

Are the conclusions drawn adequately supported by the results?

Partly

Are sufficient details of methods and analysis provided to allow replication by others?

Partly

Reviewer Expertise:

Education Technology, Applied Linguistics, Communication

I confirm that I have read this submission and believe that I have an appropriate level of expertise to confirm that it is of an acceptable scientific standard, however I have significant reservations, as outlined above.

# Reviewer report: Reviewer response for version 1

**DOI:** 10.5256/f1000research.189547.r466959

**Published:** 2026-03-30

**Authors:** Rasmitadila Rasmitadila

**Affiliations:** 1 Universitas Djuanda, Bogor, Indonesia

This article is highly relevant and innovative because it highlights the integration of AI games and deep learning in inclusive education, particularly for students with Specific Learning Disorders (SLD). It is supported by a strong and comprehensive theoretical foundation, a systematic writing structure, and in-depth analysis through a qualitative, literature-based approach, resulting in a clear and applicable conceptual framework. This article also provides practical contributions in the form of recommendations for teachers, schools, and policymakers. Several areas for improvement include the need for empirical data or field trials so that findings are not merely conceptual and can demonstrate effectiveness in real terms. The limitations of the method should not only use literature studies, which are susceptible to interpretation bias, and the still minimal discussion of technical implementation in the classroom (for example, details of the actual use of AI), so that the resulting conclusions show more potential than strong evidence.

Is the work clearly and accurately presented and does it cite the current literature?

Yes

If applicable, is the statistical analysis and its interpretation appropriate?

Partly

Are all the source data underlying the results available to ensure full reproducibility?

Yes

Is the study design appropriate and is the work technically sound?

Yes

Are the conclusions drawn adequately supported by the results?

Yes

Are sufficient details of methods and analysis provided to allow replication by others?

Partly

Reviewer Expertise:

Inclusive education and teacher development in elementary school

I confirm that I have read this submission and believe that I have an appropriate level of expertise to confirm that it is of an acceptable scientific standard.
